# Distraction Reduces Alcohol Craving: A Replication and Extension Exploring Trait Absorption and Mindfulness

**DOI:** 10.1177/29768357251384762

**Published:** 2025-10-26

**Authors:** Anthony P. De Fazio, Colby J. C. Bryce

**Affiliations:** 1 Deakin University, Burwood, VIC, Australia; 2 Worth Being Well Pty Ltd, Melbourne, VIC, Australia

**Keywords:** alcohol craving, cue-reactivity, cognitive distraction, trait absorption, trait mindfulness

## Abstract

**Background:**

Problematic alcohol use is often driven by powerful cravings elicited when individuals encounter alcohol-related cues. This cue-reactivity is a major factor in relapse and in difficulty maintaining reduced drinking.

**Aims:**

This study replicates and extends previous cue-reactivity research using a milk-based distraction task, by evaluating whether trait absorption can moderate the effect of distraction on alcohol craving. The paradigm involved exposing regular social drinkers to an alcohol-related stimulus and recording craving responses, while also assessing the influence of personality traits including absorption and mindfulness. Trait absorption and mindfulness were selected as absorption may amplify attentional focus on cues, and mindfulness supports non-reactive awareness—both factors that could moderate craving intensity and the effectiveness of distraction techniques.

**Methods:**

Seventy-nine social drinkers were recruited through convenience sampling and randomly assigned to either a control group (standard cue-reactivity paradigm) or an experimental group (cue-reactivity with a milk-based distraction task). Participants self-reported their alcohol cravings using the Visual Analogue Scale. Additionally, personality traits including trait absorption and trait mindfulness were measured to determine their predictive power on craving levels. A 3 × 2 mixed design ANOVA was used to analyse differences between groups, while hierarchical regressions assessed the role of personality traits.

**Results:**

A significant interaction between time and condition was found, indicating that participants in the distraction (experimental) group reported significantly lower alcohol cravings compared to the control group. However, the hierarchical regression analyses showed no significant effect of trait absorption or trait mindfulness in predicting changes in alcohol craving.

**Conclusion:**

Cognitive distraction (milk) significantly reduced alcohol cravings in the experimental group compared to the control group, suggesting that distractions such as those involving milk might effectively manage alcohol cravings. While personality traits including absorption and mindfulness might be less impactful in alcohol craving responses.

## Short summary

This study evaluated whether cognitive distraction reduces alcohol cravings using a cue-reactivity paradigm. Seventy-nine drinkers were exposed to alcohol-related cues, with some performing a milk-based distraction task. Results showed distraction significantly lowered cravings compared to controls. However, personality traits like absorption and mindfulness had little influence on the predictive effect of cravings.

## Introduction

Problematic alcohol use often begins with casual or social drinking and affects an estimated 400 million people worldwide.^1^ Fewer than 14% of those impacted seek appropriate care, with barriers like stigma and shame limiting access to treatment.^1^ Problematic alcohol use typically refers to patterns of drinking that result in harm to a person’s health, relationships, or ability to work, and can include criteria for Alcohol Use Disorder (AUD) as defined by the DSM-5. A significant challenge in treating problematic alcohol use and relapse following abstinence is managing cravings triggered by alcohol-related cues (cue-reactivity), which are typically unavoidable in everyday settings and often arise unexpectedly.^2^

Cue-reactivity, a key concept in addiction research, is often measured through self-report, where individuals assess their psychological and behavioural responses or cravings in response to alcohol-related stimuli (cue) compared to neutral stimuli.^3^ For example, those with alcohol use disorders display greater psychological arousal when exposed to alcohol-related stimuli, such as smelling a favourite beverage, compared to neutral stimuli like soft drinks.^2^ Research has also shown that even social drinkers exhibit high craving levels in response to alcohol cues, indicating cue-reactivity is not limited to those with alcohol use disorders.^4^ Additionally, both individuals with alcohol use disorder (AUD) and social drinkers exhibit significant psychophysiological responses to alcohol-related cues including increased heart rate, skin conductance and changes in brain activity related to the reward and stress response systems, indicating heightened autonomic arousal.^5^ These responses could suggest a robust conditioned response to alcohol cues across populations. Overall, these findings highlight that alcohol cue-reactivity is a widespread phenomenon, involving both psychological and physiological responses, and is observed across different drinking populations.

While cognitive-behavioural and mindfulness-based strategies have received significant attention in cue-reactivity craving reduction, distraction techniques remain underexplored despite their potential utility. Distraction techniques, serve as sensory or emotional diversions to alleviate cravings and withdrawal symptoms. They may fill a gap in the literature by offering a brief, accessible method to manage cravings for individuals who are not yet engaged in formal treatment or who may be deterred by stigma associated with traditional approaches. Distraction tasks, while often overlooked in formal treatment protocols, have potential as low-cost, easily implemented strategies that can help individuals manage craving episodes in real-world settings.^6^ By redirecting attention away from salient alcohol cues, such tasks may interrupt automatic cognitive and emotional processes that contribute to relapse. Embedding distraction strategies into early intervention frameworks or harm-reduction models could offer a valuable, non-stigmatising support mechanism. In this study, milk was selected as the distractor due to its rich sensory profile, including distinct taste, texture, and smell, which are theorised to more effectively engage attention than more neutral stimuli such as water,^7^ and share only weak associative ties to alcohol consumption. While milk has not been widely used in cue-reactivity studies specifically, prior research has successfully used the word ‘milk’ as a neutral thought replacement in emotional regulation tasks,^8^ supporting its application as a non-provocative distractor. Compared to alternatives like soda or juice, milk lacks common social or party-related associations, reducing the likelihood of cue overlap or unintended triggering effects.

Alongside cue-reactivity and distraction, individual differences, including personality traits, particularly how people typically focus their attention or respond to internal experiences, influence craving responses.^9^ Two traits, absorption and mindfulness, have been identified as potential predictors of craving.^10^ Trait absorption refers to a person’s capacity to become fully engrossed in internal experiences, often intensifying their emotional or physiological responses to external stimuli. Individuals with high absorption may show heightened reactivity to alcohol cues due to their tendency to deeply focus on internal imagery or sensations.^11,12^ They may therefore be more likely to fully engage with a distraction task, especially if the stimulus (eg, milk) is rich enough to hold their attention. In these cases, distraction might be more successful at pulling focus away from alcohol cues. On the other hand, people lower in absorption might not respond as strongly to the same task, making it less effective. Trait mindfulness, in contrast, involves non-judgemental awareness of the present moment and is thought to help reduce reactivity to distressing thoughts or cravings. Higher mindfulness levels may buffer against craving responses by promoting emotional regulation and attentional control.^5,13^ Rather than shifting focus to a new stimulus, more mindful individuals may instead notice cravings without reacting to them, which could mean that mindfulness and distraction work through different mechanisms. Mindfulness-based interventions (MBIs) have been shown to reduce substance use and craving, including among individuals with alcohol use disorders.^14^ These interventions typically integrate mindfulness training with relapse prevention and cognitive behavioural strategies. Although the current study focuses on trait mindfulness rather than trained mindfulness skills, the documented effectiveness of MBIs in alcohol treatment supports its inclusion as an exploratory variable. Including both traits in the study allows for a better understanding of who benefits most from distraction, and under what conditions.

This study builds on a previously published experiment which used a similar cue-reactivity paradigm and distraction task,^15^ extending it by exploring the role of trait absorption and conducting a more detailed statistical analysis in a larger and completely new, independent sample of participants. This strengthens the validity of the findings by confirming the effects of distraction on alcohol craving in a different group, enhancing generalisability beyond the original study. Incorporating trait absorption into this study is critical for clarifying for whom cognitive distraction techniques, like the milk task, might be most effective. Since absorption reflects how deeply someone can focus or get ‘lost’ in sensory experiences, it may influence how strongly a person reacts to alcohol cues and how well they can shift their attention away when distracted. If individuals high in absorption are more prone to intense cravings, understanding their response to distraction could guide more personalised interventions. Additionally, exploring absorption alongside mindfulness provides a more comprehensive view of how different cognitive and attentional traits interact with craving and coping strategies. This focus deepens theoretical insights and has practical implications for tailoring distraction-based approaches to manage alcohol cravings more effectively across diverse individuals.

The primary aim of this study is to evaluate the effectiveness of a cognitive distraction task—specifically, focussing attention on milk—in reducing alcohol cravings elicited by cue exposure. The secondary aim is to examine how individual differences in trait absorption and trait mindfulness moderate craving responses in this context. The sample comprises adult social drinkers who report regular social-based alcohol use but do not necessarily meet criteria for Alcohol Use Disorder (AUD). Participants were randomly assigned to either a control group or an experimental group, with both groups undergoing a cue-reactivity paradigm involving exposure to alcohol-related cues.

We hypothesised a significant interaction between time and condition: the control group is expected to show an increase in cravings after exposure to alcohol cues, while the experimental group is anticipated to exhibit a decrease following the milk-based distraction task. Regarding trait absorption, we hypothesised that individuals with higher levels of this trait will experience differential effects based on group assignment. In the experimental group, higher absorption was expected to enhance engagement with the milk distraction task, thereby amplifying its effectiveness in reducing cravings. Conversely, in the control group, higher absorption may lead to increased focus on alcohol cues, potentially intensifying craving responses. Although prior research has found an inverse association between trait mindfulness and craving responses,^16^ mindfulness was examined in this study as an exploratory predictor, given the brief, low-stress nature of the cue exposure paradigm, which may not have been conducive to activating mindfulness-related coping mechanisms.

## Materials and Methods

### Participants

A total of 79 participants completed the study, comprising 31 males (39%) and 48 females (61%). The mean age for male participants was 33.87 years (SD = 13.51), while for female participants it was 35.46 years (SD = 16.49). Participants reported an average age of 17 years (SD = 4.29) for their first alcoholic drink, with 24.1% indicating initiation at age 15. Regarding educational attainment, 35.4% of participants had completed an honours degree, while 1.3% had completed primary school only. Overall, 68% were university educated. In terms of occupation, 41.8% identified as professionals, and 2.5% were employed in the hospitality industry. Participants were self-identified adult social drinkers residing in Australia, recruited through convenience sampling methods, including online advertisements and university postings. Participants in the current study were not involved in any prior related research and represent a new, independent sample of social drinkers.

Inclusion criteria required participants to be at least 18 years old, report current alcohol use and provide written consent. Exclusion criteria included a current alcohol use disorder (as determined by the AUDIT) and any medical or psychiatric conditions that could interfere with study participation. A total of 79 eligible participants recruited during the data collection period met all inclusion criteria. No eligible participants were excluded, which accounts for the unusually high retention rate. Individual participant occupation, education level, age of first drink, demographics (age and gender), alcohol cravings, alcohol use, trait absorption, and trait mindfulness were measured and subsequently used in the present study.

### Materials

#### Alcohol Cravings

Participants’ alcohol craving levels were assessed using the Visual Analogue Scale (VAS), a psychometric tool for self-reported measures ranging from 0 (not at all) to 100 (extremely). Participants were asked, ‘How strong is your desire to drink alcohol for its pleasant effect?’ and ‘How strong is your desire to drink alcohol to take away an unpleasant feeling or mood?’ immediately following each cue exposure. The VAS allows participants to self-assess characteristics and attitudes in response to specific prompts, making it especially suitable for capturing rapid, momentary fluctuations in craving during cue-reactivity tasks and a standard for measuring motivational responses to alcohol cues.^17^ In this study, participants were asked to indicate their craving levels on a 100 mm line, with 0 mm representing ‘no craving’ and 100 mm representing ‘extremely’. Participants were asked to place a mark on the line that reflects how strong their craving for alcohol felt at that moment. Higher scores indicated greater craving intensity. The VAS was selected over structured craving instruments (eg, the Penn Alcohol Craving Scale) due to its sensitivity to transient, cue-induced changes in craving—a primary focus of this study. Prior research has shown that VAS measures demonstrate strong convergent validity with established craving instruments and are particularly useful in experimental paradigms requiring multiple rapid assessments.^18^

#### Alcohol Use

Participants’ alcohol-use was assessed using the World Health Organisations Alcohol Use Disorders Identification Test (AUDIT)—a 10-item self-report screening tool, that identifies individual’s risk of alcohol-related problems, including hazardous drinking, harmful alcohol use, and potential alcohol dependence.^19^ Participants were asked to respond to items such as ‘How often do you have a drink containing alcohol?’ and ‘How often during the last year have you found that you were not able to stop drinking once you had started?’ on a Likert scale ranging from 0 (‘never’ or ‘no’) to 4 (‘daily or almost daily’ or ‘yes, during the last year’). Scores were summed, with those participants who registered higher scores indicating potential alcohol problems.^20^ Descriptive AUDIT means provide value in some contexts, however the primary function of the AUDIT in this study was to apply exclusion criteria (ie, removing participants with likely alcohol use disorder). In this sense, categorical cut-offs are more informative than continuous scores for characterising our sample. Because all participants scored below the clinical threshold, the group can be validly described as social drinkers with low-to-moderate risk levels, which is the most relevant descriptor for interpreting the present findings. The AUDIT is widely used because it is brief, easy to administer, and effective across different cultures and settings.^21^ In the current study, the AUDIT demonstrated strong internal consistency (α = .88).

#### Trait Absorption

Participants’ levels of absorption were assessed using the Tellegen Absorption Scale (TAS), a 34-item self-report measure that evaluates an individual’s capacity for total engagement in sensory, imaginative, or experiential activities.^22^ The TAS identifies the degree to which individuals become fully absorbed in what they are doing, experiencing heightened emotional and sensory involvement. Participants responded to items such as ‘some of my most vivid memories are called up by scents and smells’ and ‘thoughts and images come to me without the slightest effort on my part’ using a 5-point Likert scale ranging from 1 (‘never’) to 5 (‘very often’). Scores were summed, with higher totals indicating greater levels of trait absorption. The TAS is used due to its reliability in assessing individual differences in sensory processing, imaginative experiences, and focus.^22^ In the current study, the TAS showed excellent internal consistency (α = .95).

#### Trait Mindfulness

Participants’ mindfulness levels were assessed using the Kentucky Inventory of Mindfulness Skills (KIMS), a 39-item self-report measure designed to evaluate individuals’ use of mindfulness skills in daily life.^23^ The KIMS assesses 4 key dimensions of mindfulness: Observing, Describing, Acting with Awareness, and Accepting without Judgement. Participants responded to items such as ‘I notice when my moods begin to change’ and ‘I criticise myself for having irrational or inappropriate emotions’ on a 5-point Likert scale ranging from 1 (‘never or very rarely true’) to 5 (‘very often or always true’). Scores were summed for each subscale, with higher scores indicating greater mindfulness abilities in the corresponding areas. The KIMS is widely used because it comprehensively captures various facets of mindfulness and is applicable across different settings.^24^ In the current study, the KIMS demonstrated strong internal consistency, with Cronbach’s alpha (α) = .83.

### Procedure

The study protocol was reviewed and approved by an Australian university research and ethics committee (no. HEAG-H 73_2017), ensuring compliance with ethical standards for research involving human participants. All procedures adhered to the principles outlined in the Declaration of Helsinki and relevant national regulations.

Before meeting with the researcher, participants were asked to select their preferred alcoholic beverage, which was used in the cue-reactivity experiment. Participants were scheduled to meet with the researcher at the affiliated university after 4 pm, a period of the day most associated with drinking behaviour.^25^

Participants were randomly assigned to either the control group (n = 40) or experimental group (n = 39) using block randomisation (2 blocks of 40) via randomisation.com, with seed 5295 to ensure reproducibility. All 79 randomised participants completed the study and were included in the final analyses. All participants completed a series of assessments, including the AUDIT, TAS, and KIMS. Participants urge to drink alcohol were assessed as a baseline (timepoint 1). All participants were then exposed to a neutral stimulus (water) and instructed to focus on this (timepoint 2). Importantly, to ensure that all participants had comparable alcohol cue exposure, participants in both groups were given a designated period to smell and taste the alcoholic beverage prior to the introduction of the distractor task. This approach aimed to balance cue engagement across conditions and ensure that the coping strategy (eg, distraction) was applied only after initial cue interaction. The cue-reactivity task (timepoint 3) was divided into 2 phases: (1) alcohol cue exposure and (2) distractor intervention. During the alcohol cue exposure phase, participants in both groups viewed and interacted with an alcoholic beverage while craving was assessed. In the distractor intervention phase, participants either received no milk-based instructions (control group) or were guided to focus on the taste and smell of milk (experimental group) while the alcohol was still in front of them. Timing was standardised across both phases, with each exposure and intervention lasting 3 minutes to control for potential temporal influences on craving responses.

### Statistical Analyses

Data were analysed using repeated-measures ANOVA to examine the interaction between time (baseline, post-neutral stimulus, post-alcohol cue) and condition (control vs experimental) on craving levels. Trait absorption and trait mindfulness were included as covariates to assess their moderating effects on craving responses. Post-hoc analyses with Bonferroni corrections were conducted to explore significant interactions. Effect sizes were calculated using partial eta squared (η²) to determine the magnitude of observed effects. Hierarchical multiple regression was then employed to test the unique contribution of trait absorption and trait mindfulness to craving change. Baseline craving scores were not included as covariates in the regression analyses, as baseline levels were comparable across groups (*M* = 42.53 vs *M* = 42.64). The focus of the regression models was on identifying trait-level predictors of alcohol cue reactivity at the final time point, rather than modelling within-subject change scores. In Step 1, demographic covariates (age, gender, age at first drink) were entered to control for their influence. In Step 2, trait absorption and trait mindfulness were added to evaluate their incremental predictive power. Regression models were run separately, for the control and experimental groups to directly assess whether the milk distraction manipulation altered the relationship between these traits and craving responses. This analytic approach was chosen to explore potential group-specific predictors of craving, given that the experimental manipulation (distraction) could plausibly interact with individual difference variables such as trait mindfulness and absorption. All statistical analyses were performed using SPSS, with a significance level set at *P* < .05.

## Results

Demographic data, trait absorption, and trait mindfulness were examined in Statistical Package for the Social Sciences (SPSS) for accuracy, including checks for missing values and distributional fit with multivariate assumptions. Levene’s test was non-significant, and 5 missing values were replaced using mean substitution (Little’s *MCAR* test: χ^2^ = 0.000, df = 730, *P* = 1.00). All univariate outliers fell within the acceptable range (−3.29 to 3.29), and no multivariate outliers were identified based on Mahalanobis distance (cutoff = 26.125, df = 8, *P* < .001), resulting in no participant exclusions. Consistent with the inclusion of social drinkers and exclusion of those with alcohol use disorder, participants’ AUDIT scores fell below clinical cut-offs. The distribution of scores indicated that most participants were in the low-to-moderate risk range (AUDIT < 8), aligning with other non-clinical cue-reactivity studies.^15^ While preliminary analyses comparing the control and experimental groups on baseline alcohol craving scores did not reveal significant differences, suggesting that the groups were reasonably comparable prior to the intervention.

**Figure 1.**
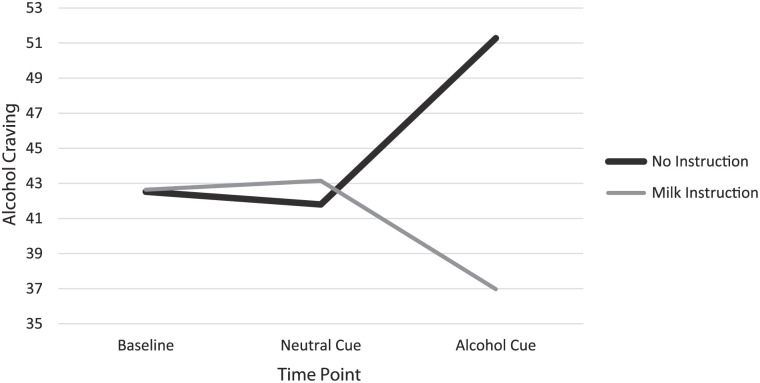
Mean alcohol-craving scores for control (no instruction) and experimental (milk instruction) groups across 3 timepoints: Timepoint 1 (Baseline), Timepoint 2 (Neutral Cue), and Timepoint 3 (Alcohol Cue).

From time point 2 (neutral cue) to 3 (alcohol cue), the control group (no instruction) reported a significant increase in alcohol craving, *M* = 9.48, SD = 17.41, *t* = 3.44, *P* < .001, and the experimental group (milk instruction) reported significant decrease in alcohol craving, *M* = −6.18, SD = 18.16, *t* = −2.13, *P* < .05 (refer to Figure 1). Means and Standard Deviations are reported in Table 1 for both control and experimental groups.

**Table 1.** Means and Standard Deviations for Urge to Drink at Baseline, Neutral and Alcohol Cue Presentations for Control and Experimental Groups.

Measure	Baseline		Neutral		Alcohol	
	*M*	SD	*M*	SD	*M*	SD
Control (no instruction)						
Alcohol craving	42.53	27.76	41.80	27.33	51.28	29.34
Experimental (milk instruction)						
Alcohol craving	42.64	29.00	43.15	29.56	36.97	26.82

### Alcohol craving measured by VAS

A 3 × 2 mixed-design ANOVA was conducted to test the hypothesis that a significant interaction effect would emerge between time point and condition. No covariates were included in this analysis as baseline craving levels were comparable between groups, and the focus was on within-subjects changes over time and between-group differences. Mauchly’s Test of Sphericity indicated a violation of sphericity assumptions (χ^2^(2) = 24.921, *P* < .001), so degrees of freedom were corrected using the Greenhouse-Geisser estimate. The analysis revealed a significant time point x condition interaction, *F*(1.56, 120.35) = 11.63, *P* < .001, ηp² = 0.131. However, no significant main effects were found for either condition, *F*(1, 77) = 0.50, *P* = .41, ηp² = 0.007, or time, *F*(2, 154) = 0.52, *P* = .550, ηp² = 0.007 (see Figure 1).

Analysis revealed a significant increase in positive urge to drink from the neutral cue to the alcohol cue presentation, *F*(1.56, 120.35) = 11.63, *P* < .001. Additionally, a significant interaction between time and condition was observed from time point 2 to time point 3, *F*(1, 77) = 15.30, *P* < .001.

A hierarchical multiple regression was conducted to test whether trait absorption would have a significant negative effect on craving responses to an alcohol cue in the experimental group and a positive association in the control group. In the experimental group, no significant relationship between trait absorption and craving was found, *b* = −0.340, *t* = −1.871, *P* = .07. Similarly, no positive association was observed in the control group, *b* = 0.15, *t* = 0.76, *P* = .45.

Another hierarchical multiple regression was conducted to test whether mindfulness would have a negative relationship with alcohol craving in response to an alcohol cue in the milk distraction (experimental) group. No significant relationship between trait mindfulness and alcohol craving was found in the experimental group, *b* = 0.022, *t* = 0.082, *P* = .94. Likewise, no significant association was found in the control group between trait mindfulness and changes in craving from baseline to post-cue exposure (*b* = –0.12, *t* = –0.68, *P* = .50), suggesting mindfulness did not predict craving levels in the absence of a distraction intervention. Refer to regression Table 2 (control group) and Table 3 (experimental group) for standardised and unstandardised coefficient statistics.

**Table 2.** Control Group: Hierarchical Regression Analysis (Time Point 1 Predictors); Age, Gender, Age at First Drink (Time Point 2 Predictors); Absorption and Mindfulness.

	Unstandardised coefficients		Standardised coefficients		
Test of significance	*B*	SE	beta	*t-value*	Sig
Step 1					
Age	−0.17	0.32	−0.09	−0.53	0.60
Gender	−2.54	9.07	−0.04	−0.28	0.78
Age at first drink	−5.65	2.51	−0.38	−2.25	0.03
Step 2					
Age	−0.12	0.35	−0.06	−0.34	0.74
Gender	−1.56	9.30	−0.03	−0.17	0.87
Age at first drink	−4.89	2.66	−0.33	−1.84	0.07
Absorption	0.15	0.20	0.12	0.76	0.45
Mindfulness	−0.21	0.31	−0.12	−0.68	0.50

Abbreviations: KIMS, Kentucky inventory of mindfulness skills; TAS: Tellegen absorption scale.

**Table 3.** Experimental Group: Hierarchical Regression Analysis (Time Point 1 Predictors); Age, Gender, Age at First Drink (Time Point 2 Predictors); Absorption and Mindfulness.

	Unstandardised coefficients		Standardised coefficients		
Test of significance	*B*	SE	beta	*t-value*	Sig
Step 1					
Age	−0.30	0.30	−0.18	-1.00	0.33
Gender	−4.40	8.62	−0.08	−0.51	0.61
Age at first drink	−0.96	0.82	−0.21	−1.18	0.25
Step 2					
Age	−0.33	0.30	−0.19	−1.10	0.28
Gender	−5.36	8.46	−0.10	−0.63	0.53
Age at first drink	−1.43	0.84	−0.31	−1.69	0.10
Absorption	−0.34	0.18	−0.32	−1.87	0.07
Mindfulness	0.02	0.27	0.01	0.08	0.94

Abbreviations: KIMS, Kentucky inventory of mindfulness skills; TAS, Tellegen absorption scale.

## Discussion

This study builds upon prior work using a cue-reactivity paradigm and milk-based distraction task.^15^ That research demonstrated that distraction (milk) could reduce alcohol craving during cue exposure and examined trait mindfulness as a potential moderator but did not explore other individual difference factors such as trait absorption, nor did it expand on the theoretical mechanisms underlying these effects. The current study extends those findings by investigating the role of individual difference factors, specifically, trait absorption in shaping craving responses. Additionally, this study deepens the theoretical understanding of how distraction might interact with attentional bias and incentive salience, while also critically evaluating limitations of trait-based measures in controlled settings. By expanding both the analytical scope and interpretive framing, this work contributes to a more nuanced understanding of distraction-based craving interventions. Results indicated that the distractor (milk) significantly reduced alcohol cravings in the experimental group, while trait absorption and mindfulness were not significantly associated with craving responses.

In the control group, which followed the standard cue-reactivity paradigm, a significant increase in alcohol craving was observed from a neutral cue (time point 2, water) to the alcohol cue (time point 3). These findings supported earlier studies indicating that pleasant sensory stimuli, including odour and sight, can enhance reward-seeking behaviours.^26^ Increased craving responses to alcohol cues are a well-documented phenomenon in both clinical and non-clinical samples; thus, observing this effect in our control group primarily serves to validate our cue-reactivity paradigm and focus attention on the novel impact of the distraction intervention. Furthermore, consistent with other research,^4^ this study confirms that social drinkers exhibit increased craving in response to alcohol cues, similar to patterns seen in alcohol use disorders. This supports the idea that attentional biases towards alcohol-related cues, which are linked to craving,^27^ are present even in social drinkers.

The significant increase in craving from a neutral cue to an alcohol cue in this study reinforces the role of attentional bias in the maintenance of alcohol consumption behaviours. This increase may also be explained by how attentional biases interact with reward-seeking behaviours. Attentional bias towards alcohol-related cues has been consistently linked to increased craving responses in previous research. Studies have shown that individuals who exhibit stronger attentional biases are more likely to experience intense cravings and, in turn, a higher risk of relapse.^27,28^ This suggests that interventions targeting attentional processes could play a critical role in craving management.

Participants in the control group, with no distractions, may have fixated on the alcohol cue due to its motivational value, intensifying cravings. The incentive salience theory suggests that alcohol cues take on heightened attention because of their association with pleasurable experiences,^29^ which likely explains the significant craving increase.

Interestingly, the experimental group showed a decrease in craving in response to alcohol cues following the distraction task. This finding suggests that distraction can reduce the dominant cognitive response by shifting focus away from the primary stimulus—in this case, the alcohol cue. The interaction observed between the experimental and control groups from time point 2 to time point 3 shows that participants in the experimental group were successfully engaged in the milk distraction task, which likely mitigated their craving responses. It is possible that the instructions associated with milk which focussed on specific sensory properties (eg, taste, texture, smell) might have contributed to its effectiveness as a distractor. These properties may have created a multi-sensory association that engaged participants, making it easier to divert their attention away from the alcohol cue. Future studies could explore whether other pleasant, neutral stimuli work similarly and whether certain characteristics of the distraction, such as sensory complexity, increase its effectiveness.

Regarding trait absorption, no significant relationship was found with craving in either group. Although trait absorption reflects a person’s general tendency to become deeply immersed in sensory or imaginative experiences, it was assessed only as a stable baseline characteristic in this study. We did not capture participants’ moment-to-moment engagement with the milk distraction task itself. Future work should include state-level measures of engagement (eg, self-reported focus during the task) to clarify how dispositional absorption interacts with actual task engagement and craving outcomes. This finding may also suggest that the participants did not engage in sufficient emotional or cognitive absorption during the task, which is a critical factor in altering craving responses.^30^ The lack of engagement may have resulted from the nature of the distraction task itself—focussing on instruction related to milk (without milk physically in front of participants) might not have been immersive enough to trigger deep absorption, as the task was relatively simple. Absorption often involves emotional involvement, and without an emotionally engaging stimulus or physical properties, participants may not have fully engaged their absorptive capacities. Additionally, external factors like environmental discomfort or lack of interest could have contributed to this lower level of engagement.

Similarly, trait mindfulness did not predict reductions in alcohol craving. This finding contrasts with literature indicating an inverse relationship between trait mindfulness and craving,^16^ particularly in the context of alcohol use. While mindfulness was included as an exploratory variable rather than a primary hypothesis, its inclusion was theory-driven and informed by prior evidence suggesting its relevance in craving regulation. While mindfulness involves sustained, non-judgemental awareness of internal experiences and attention,^31^ the distraction task used in the experimental group required participants to actively shift attention towards the neutral stimulus of milk. These distinct cognitive strategies—acceptance versus redirection—may explain why trait mindfulness did not buffer cravings in this context. Additionally, mindfulness skills often require long-term practice to be effective^32-34^ and tend to be more impactful under high-stress or emotionally charged conditions.^33,34^ In the low-stress, brief laboratory setting used in the current study, participants may not have drawn meaningfully on mindfulness-related coping mechanisms. Trait mindfulness may be more predictive of craving regulation in contexts where individuals are actively engaging mindfulness-based coping strategies, or in emotionally heightened, real-world situations.

While this study focussed on trait-level mindfulness and absorption, future research could benefit from examining these constructs as momentary states. For example, a state-level increase in absorption during alcohol cue exposure might intensify craving, especially if an individual becomes immersed in the sensory features of the cue. In such cases, distraction techniques may be particularly useful as a harm-reduction tool to interrupt this cognitive engagement. Similarly, momentary fluctuations in mindfulness may offer protective benefits in high-risk moments. Future studies using ecological momentary assessment or in-lab state measurements could help clarify how real-time changes in these processes relate to craving and response to intervention.

## Limitations

Despite the strengths of the present analysis, limitations constrain the broader interpretation of our findings. Although the sample size was adequate to detect significant within- and between-group differences—particularly for the distraction intervention—the absence of a formal power analysis limits confidence in the study’s sensitivity to detect smaller effects, especially in regression models involving trait absorption and mindfulness. This choice, combined with relatively small group sizes, may have reduced statistical power, increasing the risk of Type II errors and limiting the detection of subtle effects. Running separate regressions also increases the risk of overfitting, particularly given the large standard deviations in craving scores. While baseline craving levels were comparable across groups, they were not included in the regression models, which may limit interpretation of within-subject change. Consequently, these regression findings should be considered exploratory and interpreted with caution. The study sample size was determined primarily by feasibility and is broadly consistent with sample sizes used in prior cue-reactivity research. Although a priori power analysis was not conducted, the observed effect size for the primary time × condition interaction (ηp² = 0.131) suggests that the study was adequately powered to detect medium-to-large effects. However, it may have been underpowered to identify smaller effects, particularly in the regression models, and findings from those analyses should therefore be considered exploratory. The absence of full descriptive statistics for AUDIT scores and baseline drinking levels may have provided clearer context for characterising the sample relative to other studies. Nevertheless, all participants met inclusion criteria as social drinkers without alcohol use disorder, ensuring a reasonably homogeneous sample for the purposes of the present design.

## Future Directions

While this study focussed on treatment group differences, future research should examine how trait mindfulness and absorption independently influence craving trajectories, regardless of treatment condition. Understanding whether individuals with naturally high or low levels of these traits follow different craving patterns could clarify their role in alcohol use and inform more personalised interventions. Our findings indicate that sensory distractions, such as the milk task used here, can reduce cravings during alcohol cue exposure, highlighting potential for just-in-time interventions—digital or real-world tools that provide immediate support when cravings intensify. Smartphone apps, for instance, could prompt brief sensory distraction exercises at peak craving moments to help prevent relapse. Beyond sensory approaches, other distraction strategies—such as cognitive techniques (eg, mindfulness or thought redirection) or behavioural activities (eg, physical exercise)—may also prove effective. Comparing these distraction types could identify the most beneficial methods for different individuals or contexts, ultimately enabling tailored, real-time strategies to strengthen harm reduction efforts and support sustained control over drinking.

## Conclusion

This study extends cue-reactivity research by demonstrating that a simple sensory distraction task can attenuate alcohol cravings during cue exposure, even among social drinkers. The results support distraction as a promising just-in-time strategy to disrupt attentional bias and incentive salience processes linked to craving. Given trait absorption and mindfulness did not significantly predict craving responses, future work should examine these constructs as dynamic, state-level processes. Limitations, including modest group sizes and the absence of a formal power analysis, mean that findings, particularly from regression analyses should be interpreted cautiously. Future research should explore diverse distraction methods, compare their relative effectiveness, and integrate real-time delivery through digital or environmental prompts. Such approaches could strengthen harm-reduction strategies and support individuals in maintaining control over alcohol use.

## References

[1] World Health Organization. Alcohol. 2024. https://www.who.int/news-room/fact-sheets/detail/alcohol

[2] Glautier S Bankart J Williams A. Flavour conditioning and alcohol: a multilevel model of individual differences. Biol Psychol. 2000;52(1):17-36. 10686370 10.1016/s0301-0511(99)00022-8

[3] Carter BL Tiffany ST. Meta-analysis of cue-reactivity in addiction research. Addiction. 1999;94(3):327-340. 10605857

[4] Herrmann MJ Weijers HG Wiesbeck GA Böning J Fallgatter AJ. Alcohol cue-reactivity in heavy and light social drinkers as revealed by event-related potentials. Alcohol Alcohol. 2001;36(6):588-593. 11704627 10.1093/alcalc/36.6.588

[5] Garland EL Gaylord SA Boettiger CA Howard MO. Mindfulness training modifies cognitive, affective, and physiological mechanisms implicated in alcohol dependence: results of a randomized controlled pilot trial. J Psychoactive Drugs. 2010;42(2):177-192. 20648913 10.1080/02791072.2010.10400690 PMC2921532

[6] Ashe ML Newman MG Wilson SJ. Delay discounting and the use of mindful attention versus distraction in the treatment of drug addiction: a conceptual review. J Exp Anal Behav. 2014;103(1):234-248. 25545725 10.1002/jeab.122 PMC4410050

[7] Andrade J May J Kavanagh D . Sensory imagery in craving: from cognitive psychology to new treatments for addiction. J Exp Psychopathol. 2012;3(2):127-145.

[8] Masuda A Twohig MP Stormo AR Feinstein AB Chou YY Wendell JW. The effects of cognitive defusion and thought distraction on emotional discomfort and believability of negative self-referential thoughts. J Behav Ther Exp Psychiatry. 2010;41(1):11-17. 19716550 10.1016/j.jbtep.2009.08.006

[9] Kambouropoulos N Rock AJ. Quantifying phenomenology associated with exposure to alcohol-related cues. Imagin Cogn Pers. 2010;29(3):283-295.

[10] Rock AJ Kambouropoulos N. Toward a phenomenology of urge to drink: A future prospect for the cue-reactivity paradigm. N Am J Psychol. 2007;9(2):387-406.

[11] Sheen J Koukounas E. The role of absorption in women’s sexual response to erotica: a cognitive-affective investigation. J Sex Res. 2009;46(4):358-365. 19253136 10.1080/00224490902775843

[12] Zachariae R J⊘rgensen MM Bjerring P Svendsen G. Autonomic and psychological responses to an acute psychological stressor and relaxation: the influence of hypnotizability and absorption. Int J Clin Exp Hypn. 2000;48(4):388-403. 11011499 10.1080/00207140008410368

[13] Garland EL. Trait mindfulness predicts attentional and autonomic regulation of alcohol cue-reactivity. J Psychophysiol. 2011;25:180-189. Published online 2011. doi:doi 23976814 10.1027/0269-8803/a000060 PMC3748643

[14] Bowen S Witkiewitz K Clifasefi SL , et al. Relative efficacy of mindfulness-based relapse prevention, standard relapse prevention, and treatment as usual for substance use disorders: a randomized clinical trial. JAMA Psychiatr. 2014;71(5):547-556. 10.1001/jamapsychiatry.2013.4546 PMC4489711 24647726

[15] Koukounas E Kambouropoulos N Staiger P. The effect of cognitive distraction on the processing of alcohol cues. J Subst Use. 2019;24(6):651-654.

[16] Garland EL Boettiger CA Gaylord S Chanon VW Howard MO. Mindfulness is inversely associated with alcohol attentional bias among recovering alcohol-dependent adults. Cognit Ther Res. 2012;36(5):441-450. 10.1007/s10608-011-9378-7 PMC3532517 23280000

[17] Kambouropoulos N Staiger PK. ‘Cue reward salience’ predicts craving in response to alcohol cues. Pers Individ Dif. 2009;46(2):78-82.

[18] Ko YY Fang SC Huang WC Huang MC Chang HM. Validation of the Chinese version of penn alcohol craving scale for patients with alcohol use disorder. Psychiatry Investig. 2024;21(2):159-164. 10.30773/pi.2022.0217 PMC10910169 38433414

[19] de Meneses-Gaya C Zuardi AW Loureiro SR Crippa JAS. Alcohol use disorders identification test (AUDIT): an updated systematic review of psychometric properties. Psychol Neurosci. 2009;2(1):83-97.

[20] Reinert DF Allen JP. The alcohol use disorders identification test (AUDIT): a review of recent research. Alcohol Clin Exp Res. 2002;26(2):272-279. 11964568

[21] Reinert DF Allen JP. The alcohol use disorders identification test: an update of research findings. Alcohol Clin Exp Res. 2007;31(2):185-199. 17250609 10.1111/j.1530-0277.2006.00295.x

[22] Tellegen A Atkinson G. Openness to absorbing and self-altering experiences ("absorption"), a trait related to hypnotic susceptibility. J Abnorm Psychol. 1974;83(3):268-277. 4844914 10.1037/h0036681

[23] Baer RA Smith GT Allen KB. Assessment of mindfulness by self-report: the Kentucky Inventory of Mindfulness Skills. Assess. 2004;11(3):191-206. 10.1177/1073191104268029 15358875

[24] Moore A Malinowski P. Meditation, mindfulness and cognitive flexibility. Conscious Cogn. 2009;18(1):176-186. 19181542 10.1016/j.concog.2008.12.008

[25] Staiger PK Greeley JD Wallace SD. Alcohol exposure therapy: Generalisation and changes in responsivity. Drug Alcohol Depend. 1999;57(1):29-40. 10617311 10.1016/s0376-8716(99)00037-x

[26] Wadhwa M Shiv B Nowlis SM. A bite to whet the reward appetite: the influence of sampling on reward-seeking behaviors. J Mark Res. 2008;45(4):403-413.

[27] Field M Mogg K Bradley BP. Craving and cognitive biases for alcohol cues in social drinkers. Alcohol Alcohol. 2005;40(6):504-510. 16157608 10.1093/alcalc/agh213

[28] Field M Mogg K Mann B Bennett GA Bradley BP. Attentional biases in abstinent alcoholics and their association with craving. Psychol Addict Behav. 2013;27(1):71-80. 22905898 10.1037/a0029626

[29] Robinson MJ Robinson TE Berridge KC . Incentive Salience and the Transition to Addiction. Vol. 2. Academic Press San Diego; 2013.

[30] Wirth W Hofer M Schramm H. The role of emotional involvement and trait absorption in the formation of spatial presence. Media Psychol. 2012;15(1):19-43.

[31] Kabat-Zinn J. Mindfulness-based interventions in context: past, present, and future. Clin Psychol SciPract. 2003;10:144-156. Published online 2003. doi:10.1093/clipsy/bpg016

[32] Brown KW Ryan RM. The benefits of being present: mindfulness and its role in psychological well-being. J Pers Soc Psychol. 2003;84(4):822-848. 12703651 10.1037/0022-3514.84.4.822

[bibr33-29768357251384762] Germer CK Chan CS. Mindfulness: It’s not what you think. In: Thoma NC McKay D , eds. Working with Emotion in Cognitive-Behavioral Therapy: Techniques for Clinical Practice. The Guilford Press; 2015;11-31.

[34] Shapiro SL Brown KW Thoresen C Plante TG. The moderation of mindfulness-based stress reduction effects by trait mindfulness: results from a randomized controlled trial. J Clin Psychol. 2011;67(3):267-277. 21254055 10.1002/jclp.20761

